# A novel stepwise integrative analysis pipeline reveals distinct microbiota-host interactions and link to symptoms in irritable bowel syndrome

**DOI:** 10.1038/s41598-021-84686-9

**Published:** 2021-03-09

**Authors:** Annikka Polster, Lena Öhman, Julien Tap, Muriel Derrien, Boris Le Nevé, Johanna Sundin, Hans Törnblom, Marija Cvijovic, Magnus Simrén

**Affiliations:** 1 Department of Internal Medicine and Clinical Nutrition, Institute of Medicine, Sahlgrenska University Hospital, Sahlgrenska Academy, University of Gothenburg, 41345 Göteborg, Sweden; 2 Department of Microbiology and Immunology, Sahlgrenska Academy, University of Gothenburg, Göteborg, Sweden; 3 Danone Nutricia Research, Palaiseau, France; 4 Department of Mathematical Sciences, Chalmers University of Technology and University of Gothenburg, Göteborg, Sweden; 5 Center for Functional Gastrointestinal and Motility Disorders, University of North Carolina at Chapel Hill, Chapel Hill, NC USA

**Keywords:** Inflammatory bowel disease, Predictive medicine

## Abstract

Although incompletely understood, microbiota-host interactions are assumed to be altered in irritable bowel syndrome (IBS). We, therefore, aimed to develop a novel analysis pipeline tailored for the integrative analysis of microbiota-host interactions and association to symptoms and prove its utility in a pilot cohort. A multilayer stepwise integrative analysis pipeline was developed to visualize complex variable associations. Application of the pipeline was demonstrated on a dataset of IBS patients and healthy controls (HC), using the R software package to analyze colonic host mRNA and mucosal microbiota (16S rRNA gene sequencing), as well as gastrointestinal (GI) and psychological symptoms. In total, 42 IBS patients (57% female, mean age 33.6 (range 18–58)) and 20 HC (60% female, mean age 26.8 (range 23–41)) were included. Only in IBS patients, mRNA expression of Toll-like receptor 4 and genes associated with barrier function (PAR2, OCLN, TJP1) intercorrelated closely, suggesting potential functional relationships. This host genes-based “permeability cluster” was associated to mucosa-adjacent Chlamydiae and Lentisphaerae, and furthermore associated to satiety as well as to anxiety, depression and fatigue. In both IBS patients and HC, chromogranins, secretogranins and TLRs clustered together. In IBS patients, this host genes-based “immune-enteroendocrine cluster” was associated to specific members of Firmicutes, and to depression and fatigue, whereas in HC no significant association to microbiota was identified. We have developed a stepwise integrative analysis pipeline that allowed identification of unique host-microbiota intercorrelation patterns and association to symptoms in IBS patients. This analysis pipeline may aid in advancing the understanding of complex variable associations in health and disease.

## Introduction

Irritable Bowel Syndrome (IBS) is the most common functional bowel disorder, affecting around 10% of the general population^1–4^. It is characterized by chronic and recurrent abdominal pain in combination with altered bowel habits^1,2^. IBS strongly reduces quality of life and social function^5–7^ and leads to frequent work absenteeism^7^, resulting in a significant personal and societal economic loss^7,8^. To this day the etiology and the underlying pathophysiological mechanisms are incompletely understood, and treatment options remain sparse and suboptimal^9^. To improve this situation there is an ongoing effort to better understand the complex pathophysiology, in hopes of identifying biomarkers which can be utilized to improve diagnosis and predict treatment response^10^.

A low-grade activation of the immune system seems to be present in at least a subset of IBS patients^11–15^. This has been suggested to be of relevance for symptom generation by influencing the bidirectional communication between gut and brain (gut-brain axis) and affecting gastrointestinal (GI) sensitivity and motility, and thus leading to IBS symptoms^16^.

The gut microbiota ecosystem and its role in IBS symptom generation has been subject to extensive research in the last years, with findings in patients suggesting an altered microbiota composition^17–19^, or a potential overreaction to gut microorganisms or their metabolites^20^, as well as an influence of microbiota on the gut-brain axis^21,22^, thus contributing to both GI and psychological symptoms^23–26^.

Intestinal low-grade inflammation and microbiota alterations are hypothesized to be connected^16^, opening the question as to how exactly this host-microbiota interaction is orchestrated in the gut and how this interaction may contribute to symptom generation in IBS patients. An interaction between gut microbiota and the immune system takes place at the mucosal border, where the mucosal tissue presents a semipermeable barrier between the body and the intraluminal gut environment. Given the difficulties of culturing many of the gut microbial species, the detailed understanding of the functional interaction between host and gut microbiota is currently very limited. In silico tools that simplify the preselection of mediators with potential disease-related relevance would greatly facilitate the design and conduction of more detailed experimental analyses to elucidate how this interaction is orchestrated.

We hypothesize that the functionally related variables of this biological system exhibit an interdependence, which can be approximated by the correlations between these variables. Potentially, an integrative analysis pipeline as an exploratory method to simultaneously analyze all variables of multi-level datasets and visualize complex variable associations in order to extract relevant information, may identify which mediators involved to be most relevant in this interaction. The aim of this study was therefore to develop a stepwise integrative network analysis pipeline tailored to comprehensively analyze host-microbiota interactions and the association to key IBS symptoms. Further, we exemplify how our approach is useful for understanding relationships between gut microbiota, host barrier function and symptoms.

## Material and methods

### Study cohort and biopsy collection

A cohort of well-characterized adult patients (≥ 18 years) fulfilling the Rome III criteria for IBS^2^, were recruited at the gastroenterology outpatient clinic at Sahlgrenska University Hospital in Gothenburg, Sweden. The diagnosis of IBS was based on the clinical presentation with normal results on a limited number of investigations and tests as deemed appropriate by an experienced gastroenterologist (MS or HT). The majority of patients were referred from primary care after having already performed sufficient investigations to rule out other diagnoses. Exclusion criteria included: abnormal results on standard screening laboratory tests, other GI diseases or severe psychiatric or systemic diseases. Healthy controls were recruited through advertisement and had no history of Gl or other acute or chronic diseases and no current GI symptoms (assessed for the previous seven days). For all participants any history of drug or alcohol abuse and the inability to reliably respond to questionnaires in Swedish lead to exclusion. Any active infection (such as respiratory tract infections) also lead to exclusion, but no screening for recent infections was performed. All study participants refrained from any medication known to affect the nervous system or the GI tract during the course of this study. The use of probiotics or antibiotics was not allowed during the study period or within one month before inclusion. Informed consent was obtained for all participants in the study. They gave their written consent to participate after verbal and written information, all methods were performed in accordance with relevant guidelines and regulations and the study protocol was approved by the Regional Ethical Review Board in Gothenburg prior to the start of patient inclusion (approval number 731–09). Patients and HC were recruited as part of an extensive pathophysiological research program which included phenotyping of GI sensorimotor function, symptom profiles and mucosal functioning. Mucosal biopsies were obtained during a sigmoidoscopy (performed without prior bowel preparation) from 25–35 cm proximal of the anal sphincter using standard biopsy forceps. The biopsy specimens were taken 25–35 cm proximal of the anus during an unprepared sigmoidoscopy. Once collected, biopsy specimens were placed immediately in liquid nitrogen and stored at − 80 °C until further analysis. To verify the absence of active inflammation a routine histopathology assessment of biopsy specimens was conducted.

### Symptom assessment and IBS subtypes

To score GI symptom severity, we utilized the Gastrointestinal Symptom Rating Scale (GSRS-IBS)^27^, a validated 13-item questionnaire commonly used to assess the GI symptoms typically present in IBS. This questionnaire scores symptoms on a 7-point Likert scale from 1 = “No discomfort at all” up to 7 = “Very severe discomfort”. We have used the five GSRS domain scores (‘abdominal pain’, ‘constipation’, ‘diarrhea’, ‘satiety’ and ‘bloating’) to represent the severity of the different major IBS symptoms in our patients.

Psychological symptoms were measured using the Hospital Anxiety and Depression (HAD)^28^ scale, the Visceral Sensitivity Index (VSI)^29^ and the Multidimensional Fatigue Inventory (MFI)^30^.

The HAD^28^ measures psychological distress and was developed primarily for use in medical outpatients. It consists of 14 questions, each measuring severity with a 4-grade Likert scale (0–3). We have used these to calculate subscales for severity of anxiety (seven items) and depression (seven items), where the severity is rated on a scale from 0 to 21 with increasing severity reflected by a higher score.

The VSI^29^ contains 15 questions inquiring how patients respond to symptoms of the lower abdomen, and measures the severity of adverse responses on a 6-grade Likert scale. We have used the sum score of these items to measure the degree of GI-specific anxiety.

The MFI^30^ uses 20 question to measure different aspects of fatigue on a scale ranging from 4 to 20, with increasing severity reflected by a higher score. We have used the subscore measuring general fatigue in our analysis.

To determine IBS subtypes (Rome III)^2^, patients completed a two-week stool diary using the Bristol Stool Form scale^31^. Subtypes were based on the predominant stool consistency in concordance with the Rome III committee suggestions^2^. The subtypes IBS-M (mixed type) and IBS-U (unsubtyped) were combined into one group, IBS without predominant diarrhea or constipation, IBS-nonCnonD.

### Analysis of host gene expression

The following genes representing mucosal permeability, endocrine activity, microbial recognition and local immune-activity were assessed in the mucosal biopsies: Microbial recognition receptors (TLR 2,4,6,9; PAR2); indicators of barrier function (CLD1; MUC; OCLN; TJP1); cytokines (IL8; IL10; TNFα); granins (CHGA, CHGB, SCG2, SCG3); indicators of oxidative stress (DOUX2; NOX1); free fatty acid receptors (FFAR 2,3) and indicators of enteroendocrine function (TPH1; SLC6A4).

These markers were measured using quantitative reverse transcription PCR analysis to quantify the respective mRNA expression. To extract mRNA, the NucleoSpin RNA Kit was used according to the manufacturer’s protocol (Ref. 740,955.50, Macherey–Nagel, Düren, Germany): The biopsy was placed in lysis buffer and homogenized for two bursts lasting 2 min. Extracted mRNA was stored at − 80 °C. For quantification the mRNA was reverse-transcribed to cDNA using the “Master mix Reverse Transcription Kit” (Ref. 11,755,250, Life Technologies, Carlsbad, CA). This cDNA was added to 96-well plates containing lyophilized target probes and primers and the polymerization process was conducted using «Taqman Fast Advanced Master Mix» (Ref. 444,965, Life Technologies). To normalize the expression of the targeted genes sequence of interest, the average expression of several reference housekeeping genes (18S, POLR2A and RPLP0) was measured. The difference between the average expression of reference genes and that of the sequence of interest is given as Δ Ct (cycle threshold) and presented as 2^−(*Target-Reference*)^ or (2^Δ*Ct*^). Several housekeeping genes were used in this normalization step to minimize the chance of faulty normalization and to achieve high-quality inter-plate validation. Each analysis was run in triplicate and the triplicates averaged.

### Analysis of mucosa-adjacent microbiota

Mucosa-adjacent microbiota DNA were isolated using an adapted protocol based on Godon et al.^32^ as previously described in Tap et al.^17^. In short, amplification of hypervariable 16S rRNA regions (V5-V6) was conducted using the primers 5′-AGGATTAGATACCCTGGTA-3′ and 5′-CRRCACGAGCTGACGAC-3′. Sequencing was performed using the DNA Vision SA (Charleroi, Belgium) on a 454 Life Sciences Genome Sequencer FLX instrument (Roche) with titanium chemistry. The quality filtering and trimming of raw reads as well as OTU (operational taxonomic units) clustering and taxonomic assignment were performed using the LotuS v1.32 pipeline. If OTUs could not be assigned as detailed as genus-level, the most detailed taxonomical assignment achieved is given. Normalization for sequencing depth was performed by the median of ratios method, on the OTU table, as implemented in DESeq2^33^ in R^34^ and the abundance of 188 bacterial genera used for further analysis. Phylum-level taxonomic assignment was used for the overview-models, while taxonomic assignment up to genus-level was used for detailed analyses of the intercorrelation clusters as described in the statistical section. Phyla/genera that were screened for but showed no hits were removed from the network analysis plots.

### Data analysis

All data analyses were performed using R (version 3.4.1—“Single Candle”)^34^. Univariate comparisons were performed using Students T-test for parametric as well as Mann–Whitney U test for non-parametric data or Pearsons’ Chi^2^ test as appropriate. The network analysis in the pilot study was based on a correlation matrix of Spearman correlations to account for non-parametric data. The relative correlation strength between each variable pair determined the placement of the nodes and thickness of the edges (the connecting lines in-between), with closer approximation of nodes and thicker edges in-between reflecting a stronger correlation. The graphs were then created by plotting intercorrelations between all variables, which resulted in clustering of nodes in the cases where several variables were correlated with each other with high relative correlation strength. The network visualizations were conducted using the “qgraph”-package (version 1.4.0)^35^. Levels of α = 0.05 (*p* < 0.05) were considered statistically significant. In the pilot analysis significance values were calculated uncorrected for multiple testing to favor type 1 over type 2 error given the exploratory nature of the analysis. Both cohorts were checked for multivariate outliers using principal component analysis (with the inbuilt ‘prcomp’ function in R), and strong outliers (defined as being more than 10 units outside of the 95% confidence interval) were removed.

**Figure 1:**
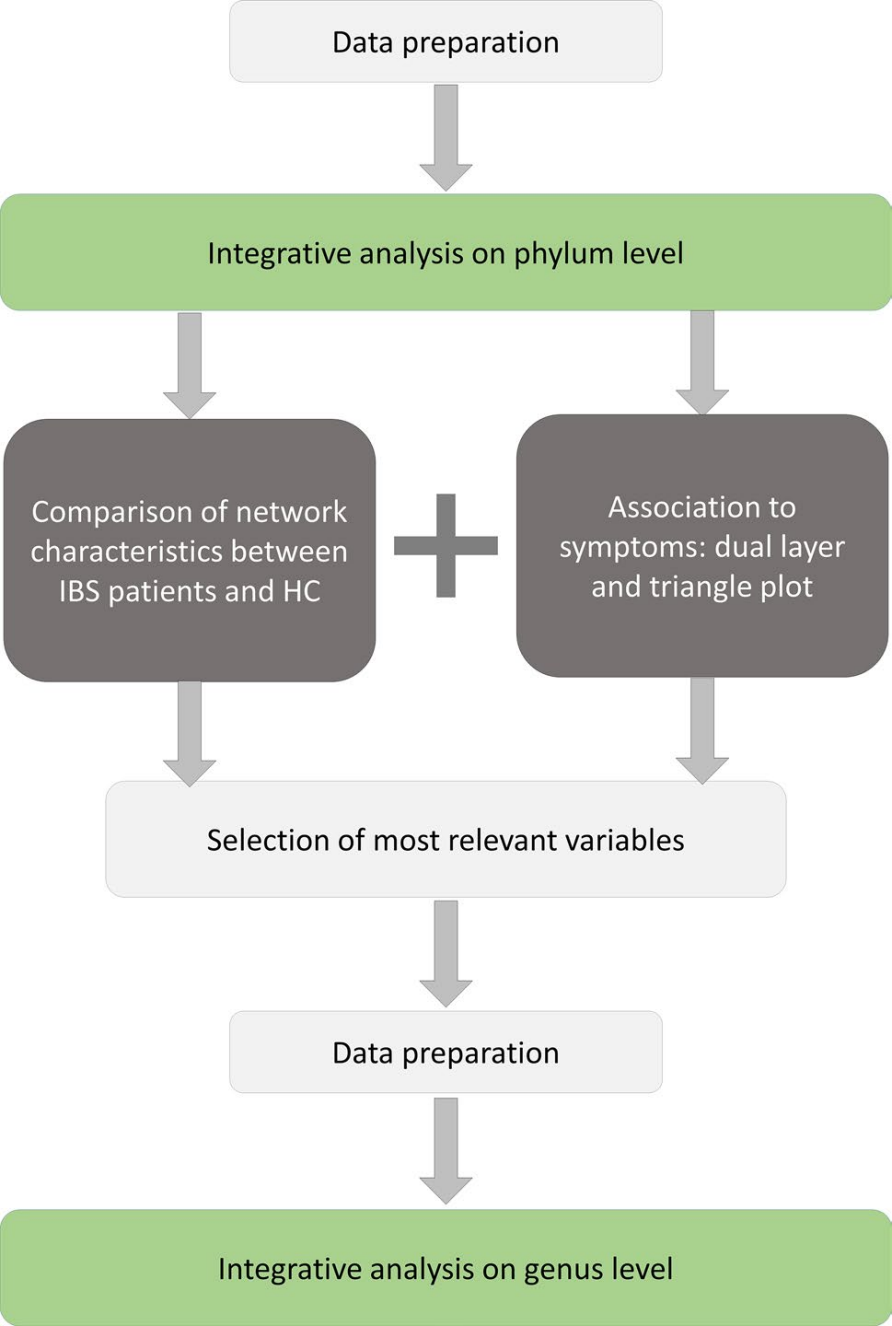
Overview of the steps performed for analyzing the pilot study dataset.

For the presented pilot study, the stepwise integrative analysis was conducted in 7 steps which are visualized in Fig. 1:

1. **Data preparation:** To limit the complexity of the initial integrative analysis an initial summarizing or clustering step is necessary. Given the limited knowledge of the physiological functioning of the gut microbial species, we here decided to use phylogenetic relatedness to summarize the bacterial genera into the respective phyla for the initial integrative analysis. As the pilot study dataset only contained a limited number of mucosal genes, these were not summarized here. The resulting datasets were quality checked using principal component analysis to test for multivariate outliers. This step was conducted with regards to the strong heterogeneity of the gut ecosystem as well as potential technical artifacts of the microbial and molecular analyses. Patients and HC were analyzed separately, and strong outliers were removed from the subsequent analysis if appropriate to reduce the risk for false-positive correlation results.
2. **Integrative analysis on phylum level:** At first all correlations between host genes and microbial phyla were plotted in their relative correlation strength, disregarding the statistical significance of the correlations, in order to create an overview plot reflecting the intercorrelation-based associations between the measures in the analyzed cohort. This step gives a graphical representation of interconnectedness of the variables entered into the model and was used to identify clusters of close intercorrelation between markers of mucosal activity and microbiota. Secondly, the same correlation matrix was plotted, but statistically insignificant correlations (p ≥ 0.05) were omitted from the plot. This step was conducted especially with regards to exploring the statistical significance of the before identified intercorrelation clusters.
3. **Comparison of network characteristics between IBS patients and HC:** The plots derived from step 2 were compared between patients and HC regarding network patterns/clusters as well as network density and differences in network patterns highlighted.
4. **Association to symptoms:** Here three separate network plots were created, representing (i) associations between microbiota and host genes, as performed in step 2, (ii) associations between GI symptoms and (iii) associations between psychological symptoms. Statistically significant correlations between variables of these plots were then added to create the respective figures.
5. **Selection of most relevant variables:** Steps 3 and 4 were used to select variables that were part of patient-specific network patterns and/or significantly correlated to symptoms.
6. **Data preparation:** In step 5 noteworthy phyla were selected, which were now split up again into the respective genera, to achieve a more detailed understanding of which specific genera were associated to the respective mucosal genes. Another quality check was performed as described in step 1.
7. **Integrative analysis on genus level:** Previously identified relevant mucosal genes and associated microbiota were plotted and associations tested for statistical significance.

Likewise, microbiotas associated only to clinical symptoms were plotted and associations tested for statistical significance.

## Results

### Development of an integrative analysis pipeline

**Figure 2:**
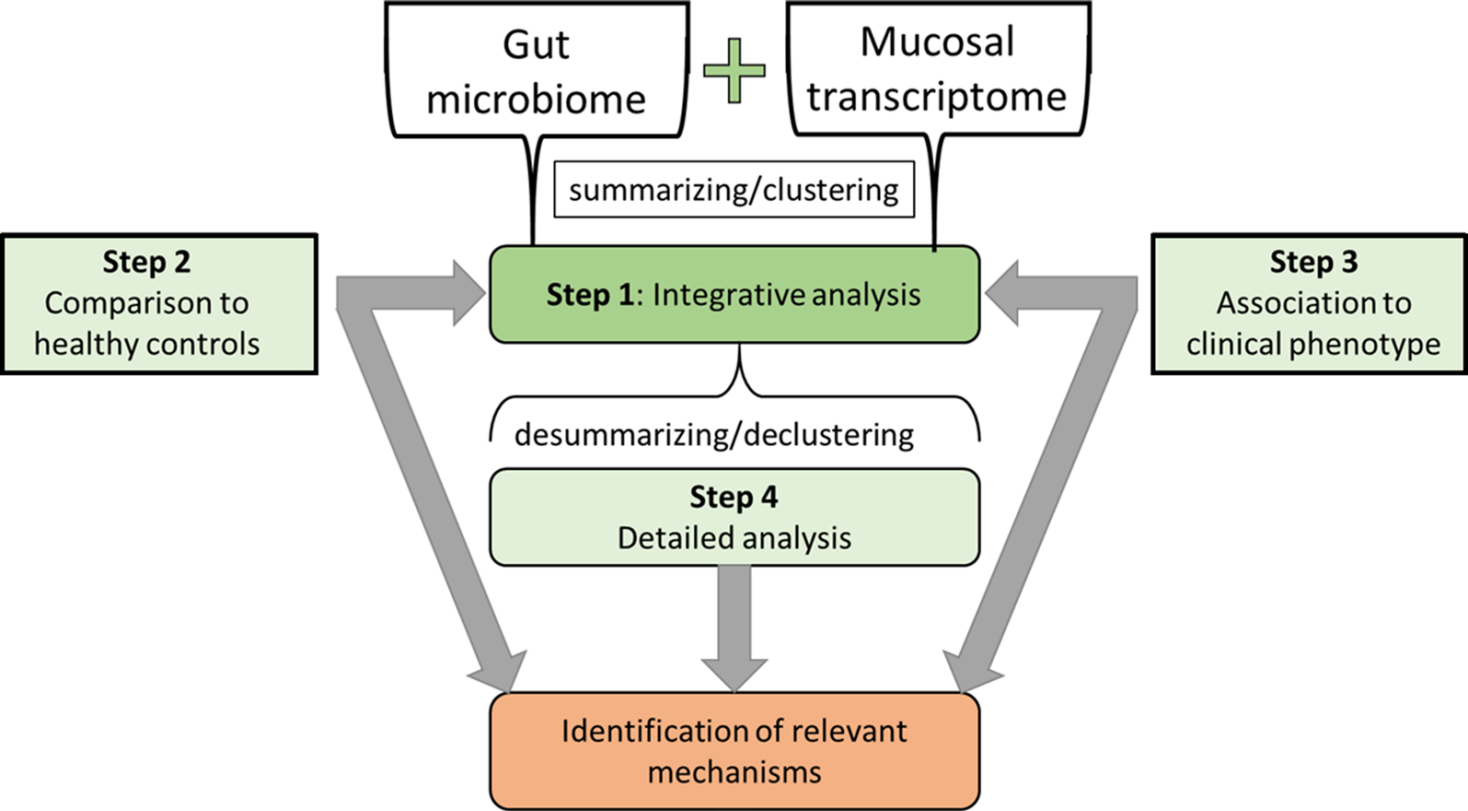
Concept of the stepwise integrative analysis pipeline.

This section presents the developed integrative analysis pipeline, whereas the execution of the steps in the pipeline on the dataset, and the results thereof, are described in the consecutive sections. A conceptual visualization of the integrative analysis pipeline is given in Fig. 2. Thus, variables A (for example gut microbiome) and variables B (for example host gene expression) are initially combined into summary-variables that are then associated to each other in an integrative analysis, and plotted in a network graph. The initial summarizing step is relevant to enhance the visibility of patterns in the network graph. This is done separately in both patients and HC, and are then compared between the two groups. Furthermore, the patient network is associated to variables representing the clinical phenotype, such as key symptoms. By comparing patients to HC and by associating to the clinical phenotype, summary-variables of high interest are identified. These specific summary-variables are then de-summarized to identify in detail which variables are directly associated.

### Description of the study cohort

**Figure 3:**
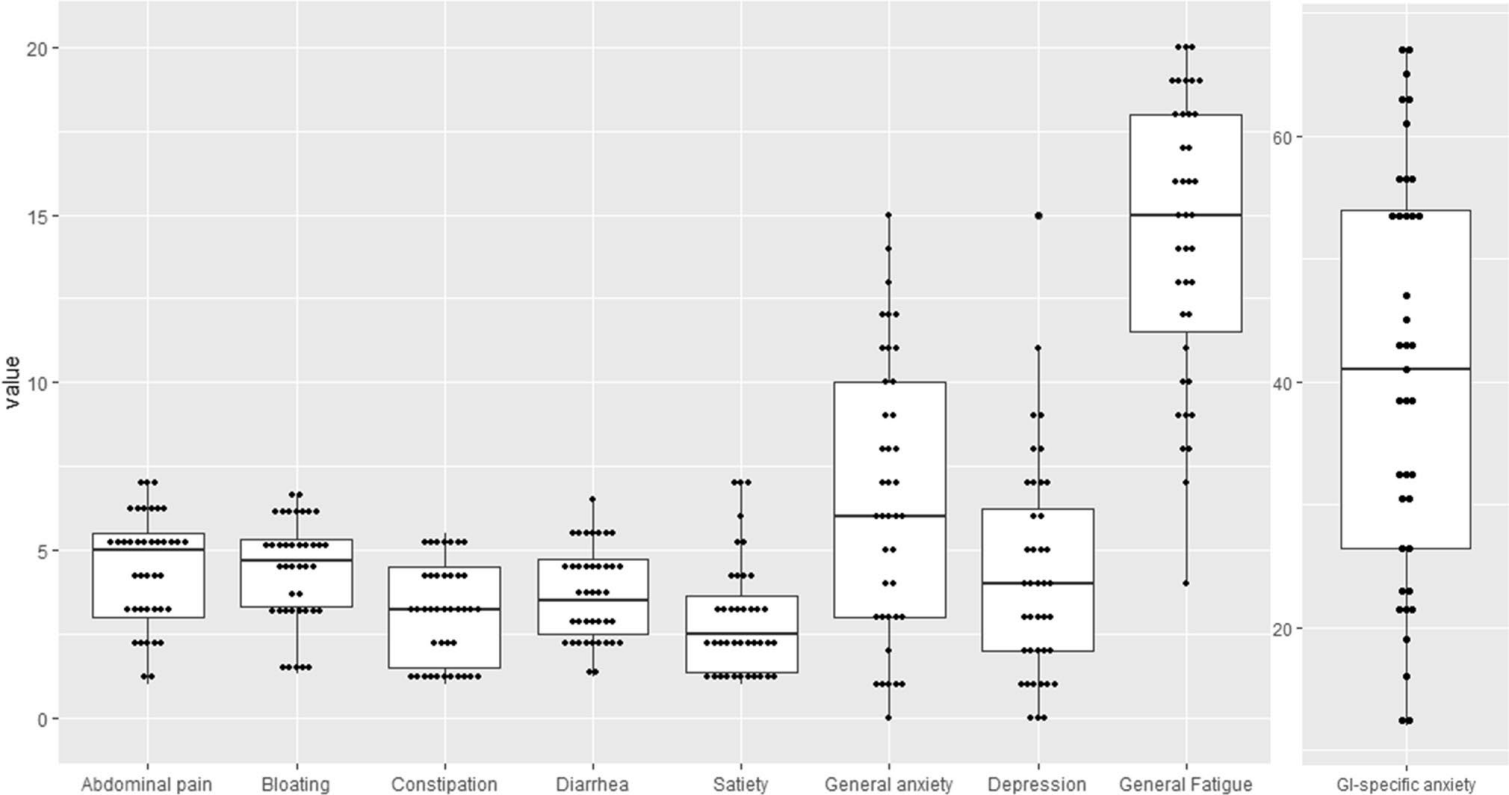
Distribution of symptom severity.

The gender distribution among IBS patients and HC was similar, but the mean age was higher in the IBS group. Most patients had moderate symptom severity as measured by the GSRS subscores with the median of the respective scores ranging from 2.5 (satiety) to 4.7 (bloating) (total range of GSRS scores 1–7), and Rome III subtypes were present to a similar extent. Details are shown in Table 1 and Fig. 3.

**Table 1:** Demographics, Rome III subtypes and symptom severity.

	Patients		Healthy controls		*p* value
Gender female/male	24/18		12/8		
Age mean (range)	33.6	(18–58)	26.8	(23–41)	0.6^a^
IBS-C	13	31%			0.02^b^
IBS-D	15	35.7%			
IBS-nonCnonD	14	33.3%			
Abdominal pain (GSRS) Median (IQR)	5	(3–5.5)			
Constipation (GSRS) Median (IQR)	3	(1.3–4.5)			
Diarrhea (GSRS) Median (IQR)	3.5	(2.5–4.8)			
Bloating (GSRS) Median (IQR)	4.7	(3.3–5.3)			
Satiety (GSRS) Median (IQR)	2.5	(1–3.8)			
General anxiety (HAD) Median (IQR)	6	(3–10.5)			
Depression (HAD) Median (IQR)	4	(1.5–6.5)			
GI-specific anxiety (VSI) Median (IQR)	42	(26.3–55.5)			
General fatigue (MFI) Median (IQR)	15	(10.3–18)			

GSRS: gastrointestinal symptom rating scale.

HAD: Hospital anxiety and depression scale.

VSI: Visceral sensitivity index.

MFI: multidimensional index.

IQR: Interquartile range.

^a^chi^2^-test, ^b^student’s t-text.

The averaged expression levels of the mucosal host genes did not differ between IBS patients and HC. However, in IBS patients significantly less OTUs were assigned to unclassified Bacteria, to Lentisphaerae and Firmicutes (all *p* < 0.01). Expression of mucosal host genes as well as normalized microbiota OTU counts are shown in Supplementary Figures 1 and 2.

### Application of the integrative analysis of host-microbiota interactions

**Figure 4:**
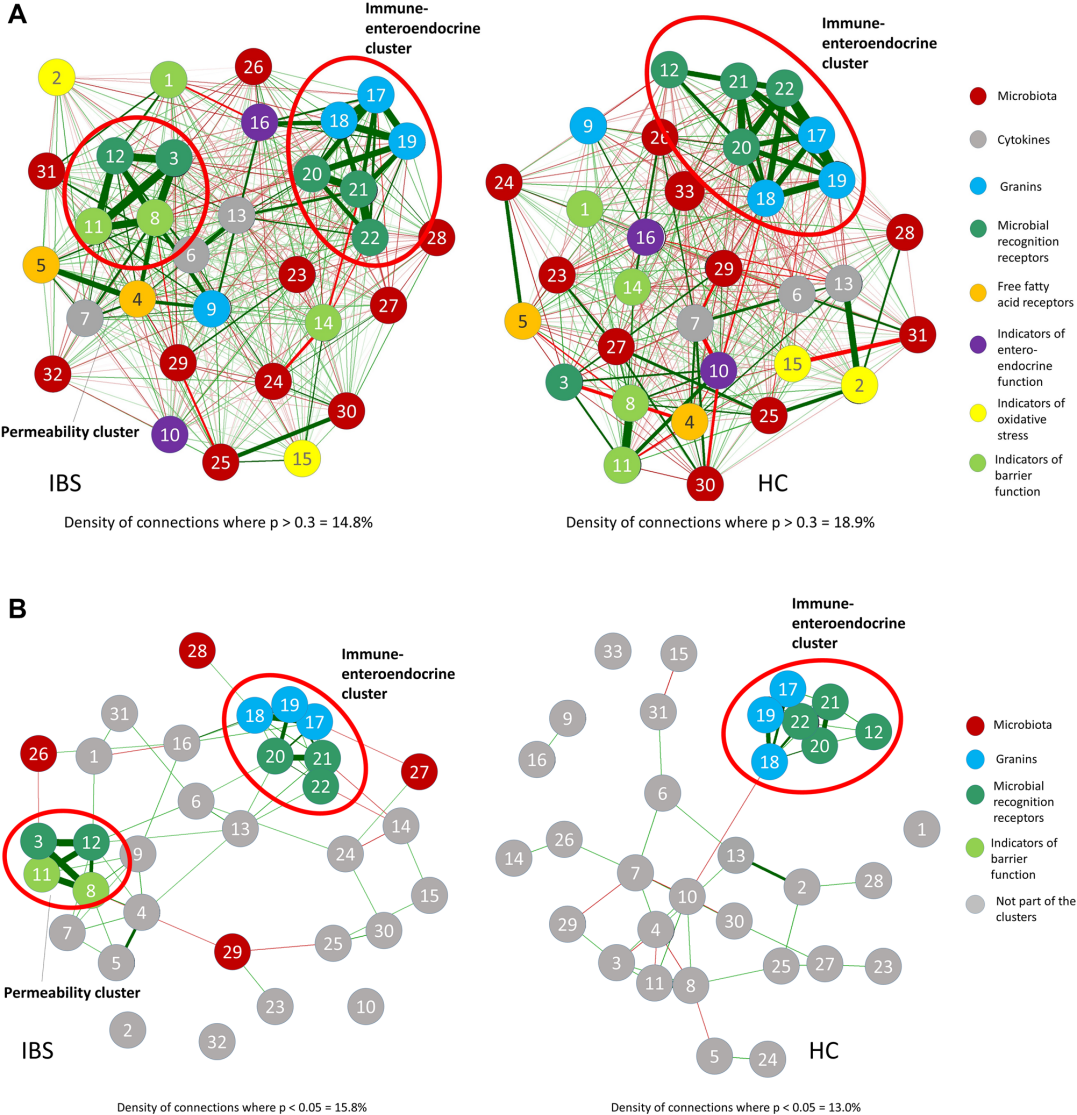
Integrative analysis on phylum level. (**A**) Overview, all correlations are plotted in relative correlation strength (disregarding statistical significance) to understand the relationships between the variables present in the given cohorts. Clusters of high intercorrelation are marked with a red circle. (**B**) Significance test: Only statistically significant correlations are plotted to estimate the generalizability of the patterns. Highlighted in color are the intercorrelation clusters. Clusters of high intercorrelation are marked with a red circle. Green edges show positive correlation, red edges negative correlation. Closer proximity of nodes and thicker edges show higher relative correlation strength. 1 = CLD1; 2 = DOUX2; 3 = PAR2; 4 = FFAR2; 5 = FFAR3; 6 = IL10; 7 = IL8; 8 = OCLN; 9 = SCG2; 10 = SLC6A4; 11 = TJP1; 12 = TLR4; 13 = TNFα; 14 = MUC; 15 = NOX1; 16 = TPH1; 17 = CHGA; 18 = CHGB; 19 = SCG3; 20 = TLR2; 21 = TLR6; 22 = TLR9; 23 = unclassified bacteria; 24 = Actinobacteria; 25 = Bacteroidetes; 26 = Chlamydiae; 27 = Firmicutes; 28 = Fusobacteria; 29 = Lentisphaerae; 30 = Proteobacteria; 31 = Tenericutes; 32 = Verrucomicrobia; 33 = Euryarchaeota.

The mucosal host-microbiota interaction model in IBS patients and HC based on intercorrelation of all variables is visualized in Fig. 4. The network density, describing the number of actual connections in relation to the number of possible connections was higher with regards to correlation strength in the HC network, defined as density of connections where *p* > 0.3 (IBS = 14.8% vs. HC = 18.9%) (Fig. 4A). The IBS patients’ network however showed a higher density with regards to statistically significant connections, defined as density of connections where *p* < 0.05 (IBS = 15.8% vs. HC = 13.0%) (Fig. 4B). Two outliers were removed in the PCA step.

All correlation coefficients of the two models are given in Supplementary Figures 2 and 3.

Uniquely in patients, a cluster of four host genes associated to barrier function and microbial recognition was identified, referred to as the ‘*permeability cluster*, which was associated to the phyla Chlamydiae and Lentisphaerae (Fig. 4 A and B). In both patients and HC, a cluster consisting of secretogranins and chromogranins as well as microbial recognition receptors was identified (six genes in IBS and seven in HC), referred to as the ‘*immune-enteroendocrine clusters*’. In patients the latter cluster was associated to the phyla Firmicutes and Fusobacteria, whereas the HC cluster showed no statistically significant association to microbiota at phylum level (Fig. 4 A and B).

The permeability cluster uniquely found in patients contained occludin (OCLN) and tight junction protein 1 (TJP1), both involved in epithelial barrier function, as well as the microbial recognition receptors Toll-like receptor 4 (TLR4) and protease activated receptor 2 (PAR2), which were all positively correlated to each other. The negative correlation of this cluster to the phyla Chlamydiae and Lentisphaerae was statistically significant (Fig. 4B).

In both IBS patients and HC, the immune-enteroendocrine cluster contained the Toll-like receptors TLR-2, TLR-6 and TLR-9 and the chromogranins A and B as well as secretogranin 3, in HC additionally TLR-4. In patients this cluster was negatively correlated to mucosa-adherent Firmicutes, and positively correlated to Fusobacteria, whereas in HC no statistically significant association to any microbiota was confirmed (Fig. 4B).

### Association between pattern of host-microbiota interactions to symptoms

**Figure 5:**
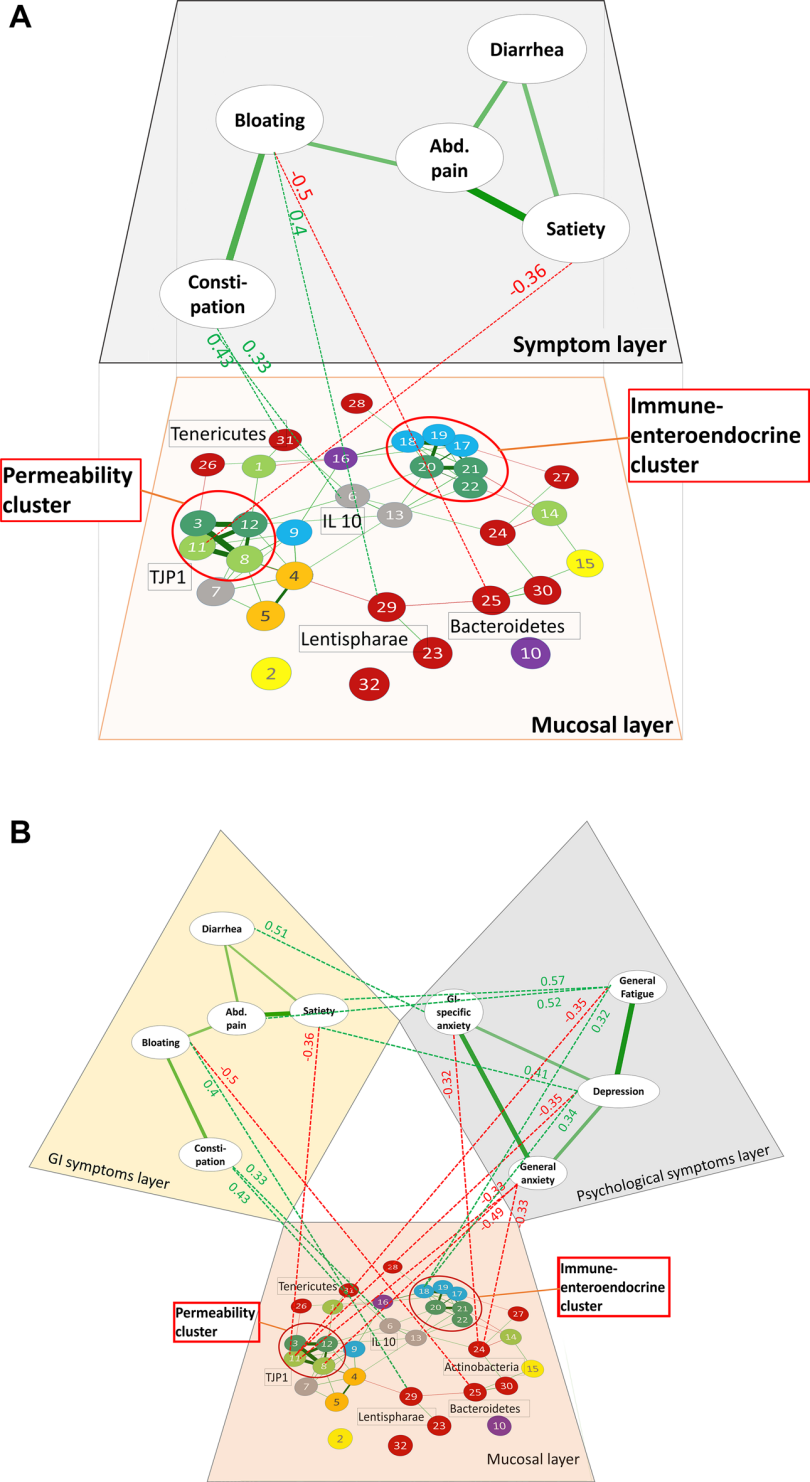
(**A**) Dual layer visualization of the association of host genes and mucosa-adherent microbiota with GI symptoms. The GI symptom layer reflects the relationship of the key IBS symptoms abdominal pain, constipation, diarrhea, satiety and bloating with each other, while the mucosal layer reflects patters of host-microbiota interaction corresponding to Fig. 3. Between-layer correlations are dotted and rho-value is given. (**B**) Triangle plot integrating three layers to show the inter-layer associations between patterns of host-microbiota interaction, GI symptoms and psychological symptoms. Between-layer correlations are dotted and rho-value is given. Only statistically significant correlations are plotted. Green edges show positive correlation, red edges negative correlation. Closer proximity of nodes and thicker edges show higher relative correlation strength in the two layers, which is disregarded in the between-layer edges. 1 = CLD1; 2 = DOUX2; 3 = PAR2; 4 = FFAR2; 5 = FFAR3; 6 = IL10; 7 = IL8; 8 = OCLN; 9 = SCG2; 10 = SLC6A4; 11 = TJP1; 12 = TLR4; 13 = TNFα; 14 = MUC; 15 = NOX1; 16 = TPH1; 17 = CHGA; 18 = CHGB; 19 = SCG3; 20 = TLR2; 21 = TLR6; 22 = TLR9; 23 = unclassified bacteria; 24 = Actinobacteria; 25 = Bacteroidetes; 26 = Chlamydiae; 27 = Firmicutes; 28 = Fusobacteria; 29 = Lentisphaerae; 30 = Proteobacteria; 31 = Tenericutes; 32 = Verrucomicrobia; 33 = Euryarchaeota.

Two plots showing the between-layer relationship of key IBS symptoms (symptom layers) and the intercorrelation patterns of host-microbiota interaction (mucosal layer) were created to highlight variables significantly associated to the clinical phenotype of the IBS patients. The between-layer correlations predominantly demonstrated an association between psychological symptoms and the permeability cluster as well as the immune-enteroendocrine cluster, whereas gastrointestinal symptoms were directly associated to several microbiota and mucosal genes, and indirectly connected to the clusters (Fig. 5 A and B). All significant correlations between symptoms and microbiota or host genes were of moderate strength ranging from ρ =|0.33| – |0.5|.

Associations between the GI symptom layer and the mucosal layer are featured in Fig. 5 A. Specifically, bloating showed a positive correlation to Lentisphaerae, as well as a negative correlation to Bacteroidetes; constipation was positively associated to Tenericutes as well as the anti-inflammatory cytokine IL-10; satiety showed a negative correlation to the tight junction protein TJP-1. However, abdominal pain and diarrhea showed no significant correlation to the analyzed host genes or microbiota.

The associations of both the mucosal layer and the GI symptom layer with the psychological symptoms layer are visualized in Fig. 5 B. General anxiety (HAD) was negatively correlated to Actinobacteria, and the tight-junction proteins OCLN and TJP1, but showed no statistically significant direct correlation to the GI symptom layer. Depression was also negatively correlated to TJP1, and positively correlated to enteroendocrine protein CGB, and positively correlated to satiety. GI-specific anxiety (VSI) showed a negative correlation to Actinobacteria and a positive correlation to diarrhea. General fatigue (MFI) showed a positive correlation to CGB, and a negative correlation to TJP1, and was positively correlated to abdominal pain and satiety.

### Detailed taxonomic assignment of patient-specific interaction patterns on genus level

**Figure 6:**
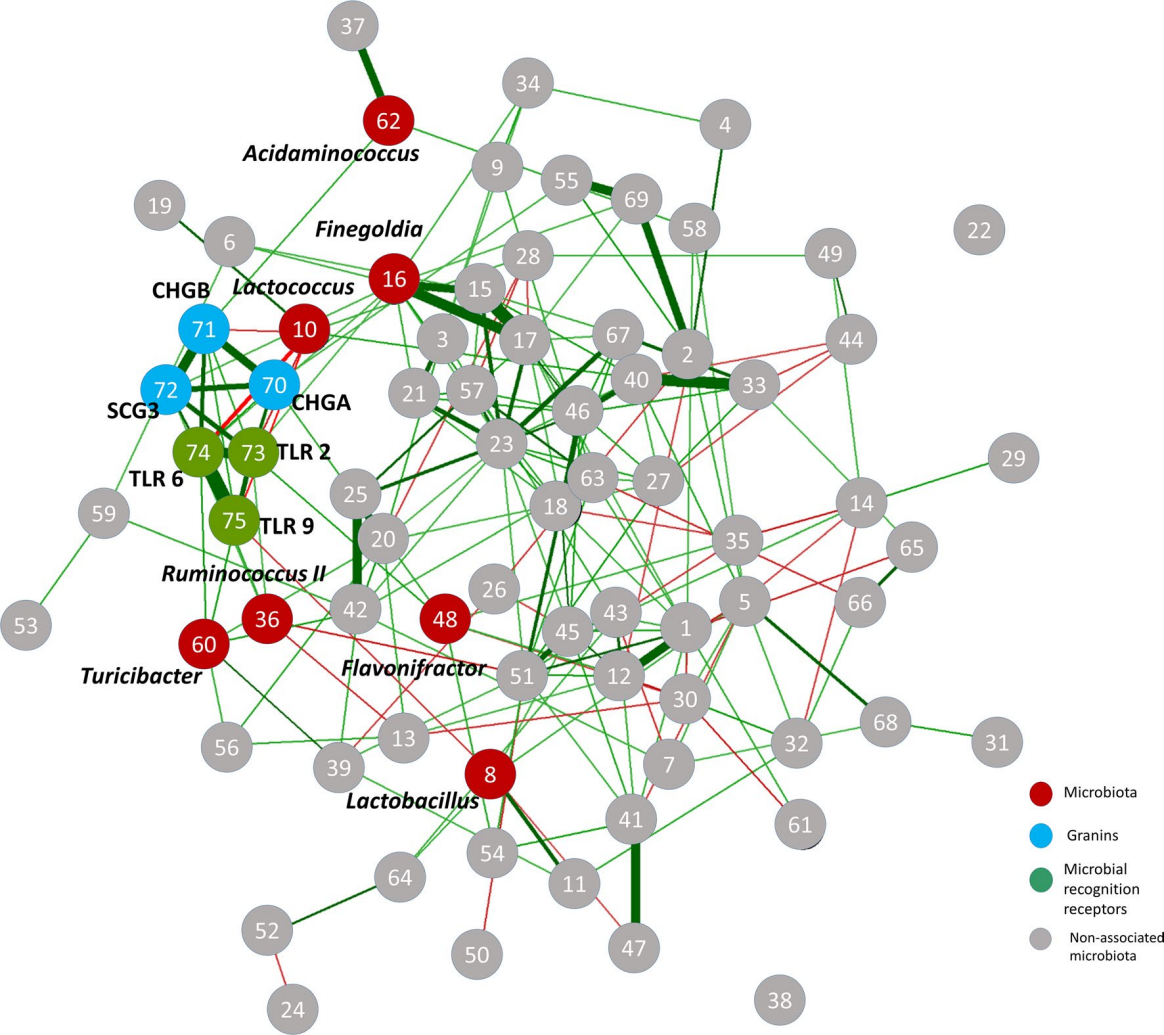
Detailed analysis of the immune-enteroendocrine cluster in IBS patients and microbiota on genus level. Red color highlights the bacteria significantly associated to mucosal markers, while grey marks remaining bacteria of the phylum. Only statistically significant correlations are plotted. Green edges show positive correlation, red edges negative correlation. Closer proximity of nodes and thicker edges show higher relative correlation strength. Most relevant nodes are labelled in the figure, and the legend to the grey nodes is given in supplementary.

To better understand the above described associations, we performed additional network analyses with a more detailed taxonomic assignment on genus level (“desummarizing step”, as described in Fig. 2). This was done on the variables contained in the patients’ immune-enteroendocrine cluster as well as bacteria of the Firmicutes-phylum, since after exclusion of outliers, Fusobacteria showed no statistically significant association. This showed that the genes of the immune-enteroendocrine cluster of patients were positively correlated to *Finegoldia, Peptoniphilus, Ruminococcus II* (of the family Lachnospiracea)*, Flavonifractor, Turicibacter and Acidaminococcus*, and negatively correlated to *Lactobacillus* and *Lactococcus* (Fig. 6). All correlation coefficients are given in Supplementary Figure 5.

No further detailed analyses were conducted with regards to the permeability cluster in IBS patients since only the family Parachlamydiaceae of the Chlamydia phylum and only the genus *Victivallis* of the Lentisphaerae phylum were found to be present in the samples. Likewise, since the immune-activation cluster in HC showed no significant association to any microbiota, no detailed analyses were performed.

Additionally, genus-level network analyses were performed on Actinobacteria and Bacteroidetes, which were associated to symptoms but not mucosal genes. No further analyses were performed on the genera Tenericutes and Lentispharae, as for Tenericutes only *Anaeroplasma* and *Asteroleplasma*, and for Lentispharae only the genus *Victivallis* were found.

The Actinobacteria *Microbacterium, Rothia, Bifidobacterium* and *Gardnerella*, as well as unclassified genera from the families Bifidobacteriaceae and Coriobacteriaceae were associated to GI-specific and psychological symptoms (Supplementary Figure 3). All correlation coefficients are given in Supplementary Table 6.

The Bacteroidetes genera *Barnesiella*, *Paraprevotella*, *Prevotella*, *Alistipes* and *Pedobacter* as well as unclassified genera from the families Prevotellaceae and Flavobacteriaceae were negatively associated to both GI-specific and psychological symptoms (Supplementary Figure 3). All correlation coefficients are given in Supplementary Table 7.

## Discussion

In this exploratory study we developed a stepwise integrative analysis pipeline tailored to identify host-microbiota interactions and link to symptoms. Furthermore, to demonstrate the execution, we applied the pipeline to a pilot data set. Here we identified patterns of host-microbiota interactions found in IBS patients but not in HC, and were able to link these patterns to IBS symptoms, thus confirming the usefulness of our approach. Intestinal bacteria may regulate intestinal epithelial barrier function and the immune response, and previous studies support the potential relevance of our findings. For example, Lactobacillus strains have been demonstrated to induce TLR2 signaling and impact tight junction proteins in epithelial cells^36^. Further, L. plantarum has been shown to induce genomic DNA-dependent and TLR9-mediated elafin secretion in an epithelial cell line^37^.

This study demonstrates a novel approach to explore and compare complex variable interrelatedness in both IBS patients and HC and to elucidate mucosal crosstalk between host and microbiota. This approach utilizes the correlation between variables to estimate potential interrelatedness. The resulting correlation patterns need to be interpreted with caution, but may nevertheless highlight variables which might be functionally connected, either directly or indirectly by mediators. Therefore, using correlations as an approximation for the potential functional connection of variables may aid in the development of novel hypotheses regarding IBS pathophysiology and symptom generation, and suggest relevant targets for further experimental exploration. The differences of correlation patters of host-microbiota interaction found between IBS patients and HC, and the association of these to key IBS symptoms, validate the suitability of our stepwise integrative analysis pipeline to analyze complex, multilevel datasets and define which variable/s may be of most relevance. The multilayer graphs provide further insight and enhance interpretability of variable association and the role of potential mediators in complex diseases such as IBS.

Importantly, we demonstrated some patterns to be common for both IBS patients and HC, whereas others were specific for patients. Potentially, patient-specific patterns are related to disease mechanisms and may improve understanding underlying pathology. In addition, the visualization strategy presented here can be scaled to include additional compartments, thus facilitating the reduction of the amount of latent variables and increasing the resolution of potential pathomechanistic insights. Thus, the integrative analysis pipeline developed and presented in this study can provide additional information that traditional univariate analyses do not reveal, making this method useful for research questions relevant to the field of multifactorial diseases.

A method that aids in identifying relevant variables in a highly complex dataset is especially relevant for improving our understanding of the details of host-microbiota interactions. The majority of gut microbiota species are difficult and time-consuming to culture, limiting our experimentally validated knowledge of the physiological function of these species. Integrative analyses as demonstrated here may therefore be highly useful to identify which of these microbial species are most relevant to focus on experimentally, improving the cost-effectiveness of such studies and likelihood of relevant findings.

In this study we have preceded the network analysis with a screening for multivariate outliers. This was done for two reasons: The in between sample variability expected in microbiota analyses as well as the heterogeneity typically observed in IBS cohorts^9,38^. A few microbial genera were only present in singular individuals, the cause of which is yet unidentified. Given the relatively low number of subjects included and the exploratory nature of this study, excluding these outliers likely is the best compromise to reduce spurious results while maximizing statistical power. In studies including a higher amount of study subjects this problem may additionally be addressed by applying a cut-off that excludes microbial genes that are present only in very low frequencies or only in a very low number of individuals, thus further reducing technical and biological noise in the dataset.

A major challenge in integrative analyses is the question of how to quantify the association between variables given highly diverse data characteristics. Rank correlations, as applied in this study, enable the simultaneous analysis of data of different scales. As mentioned above, correlations need to be interpreted with caution. In biological systems functionally connected mechanisms are commonly correlated, but, given the complexity of biological systems, direct causality cannot be concluded. Nevertheless, this integrative analytical approach can be applied to narrow down which components of complex biological systems may most likely be functionally connected and therefore are most relevant targets for more detailed experimental analyses. Furthermore, by applying a multilayer approach to the research question, the role of potential mediators is visualized and can be taken into account when interpreting the plots. Additional layers can be added to the analysis in future studies to further enhance the complexity of the obtained results.

Our stepwise integrative analysis pipeline comprises several steps, which, in combination, narrow down the most relevant variables. The initial step is a summarizing/clustering step, which favors the interpretability of the resulting integrative plot, but sacrifices detailed information. Therefor complementary secondary integrative plots are created to achieve a higher resolution of variable associations. Choosing the right strategy for creating the initial summarized variables remains a challenge since very little is known about most gut microbial species. With growing knowledge about the physiological function of these species a better summarizing criterion than phylogenetic relatedness may be developed, but at this time point this likely is the best alternative. Despite these considerations, our approach showed very noteworthy results in the presented study, suggesting that future studies, with further improved summarizing step to reduce the risk of false-negative findings, may yield even more accurate results.

There are several limitations to this study. First, the rather small number of mucosal genes analyzed in this study is a limitation, as genes not analyzed in this study will most likely also be of high relevance in the orchestration of host-microbiota interaction. Hence, using comprehensive methods such as whole transcriptome shotgun sequencing to obtain a more extensive dataset of host gene expression would make a more detailed and accurate identification of potential key players possible. Second, while 16 s sequencing is state-of-the-art in microbial research and useful in identifying the microbial composition in obtained samples^39^, it does not provide information of quantitative nature. Third, traditionally OTU counts are normalized to account for sequencing depth using ratio-calculations or rarefication^40^. These methods have been criticized, and currently various improved methods are developed, tested and discussed^40–42^. We have thus chosen to apply the median of ratios method as implemented in DESeq2^33^, which has shown to produce more reliable results when normalizing count data than traditional methods^40,42^. Fourth, our patients were recruited from a secondary/tertiary care unit, which holds the risk of a recruitment bias leading to higher severity of symptoms. Fifth, the microbiota data was sequenced using only the V5-V6 regions, limiting the resolution of this data, which may be the reason a number of bacteria remained unclassified. Still, this approach has so far been one of the most used methods to analyze gut microbiota. Despite these limitations, we were able to show noteworthy differences between well phenotyped IBS patients of all subtypes and HC and identify distinct associations between host genes and microbiota, supporting the generalizability of our findings. The extensive phenotyping conducted on our participants allows us to assume a low degree of biological noise in our dataset, which is a strong argument for the relevance of our results.

Taken together, we have developed a novel strategy tailored for analyzing the complex host-microbiota interaction and its association to symptoms and demonstrated its successful application to a pilot study dataset. Distinct differences between IBS patients and HC were identified, and an association of host-microbiota interaction patterns to key IBS symptoms was demonstrated, as well as the potential role of mediators highlighted. Applying the described pipeline to even more comprehensive datasets may aid in unravelling the complex host-microbiota interactions in health and disease and aid in identifying key pathophysiological mechanisms and potential biomarkers. The approach can be used to screen various complex datasets and identify the most relevant variables for further experimental validation.

## Supplementary Information

Supplementary Information

## Abbreviations

IBS: Irritable bowel syndrome
GI: Gastrointestinal
IBS-C: IBS with constipation
IBS-D: IBS with diarrhea
IBS-M: IBS with mixed loose and hard stools
IBS-U: Unsubtyped IBS
IL: Interleukin
TJP: Tight junction protein
TLR: Toll-like receptor
TNF: Tumor necrosis factor
OCLN: Occludin
TPH 1: Tryptophan hydroxylase 1
PAR2: Protease activated receptor 2
CLD: Claudin
MUC: Mucin
CHG: Chromogranin
SCG: Secretogranin
DOUX: Dual-oxidase
NOX: NAD(P)H oxidase
FFAR: Free fatty acid receptor
SLC: Solute carrier
