# Supplementary material for: A novel stepwise integrative analysis pipeline reveals distinct microbiota-host interactions and link to symptoms in irritable bowel syndrome
Source: Sci Rep. 2021 Mar 9;11:5521. doi: 10.1038/s41598-021-84686-9 (PMC7943560; doi:10.1038/s41598-021-84686-9)
Supplement: Supplementary file 1 — Supplementary Information [file 41598_2021_84686_MOESM1_ESM.docx]

Supplementary material to the article

A novel stepwise integrative analysis pipeline reveals distinct microbiota-host interactions and link to symptoms in Irritable Bowel Syndrome

Annikka Polster^1^, Lena Öhman^1,2^, Julien Tap^3^, Muriel Derrien^3^, Boris Le Nevé^3^, Johanna Sundin^1,2^, Hans Törnblom^1^, Marija Cvijovic^4^*, Magnus Simrén^1,5^*

# Supplementary Figure 1


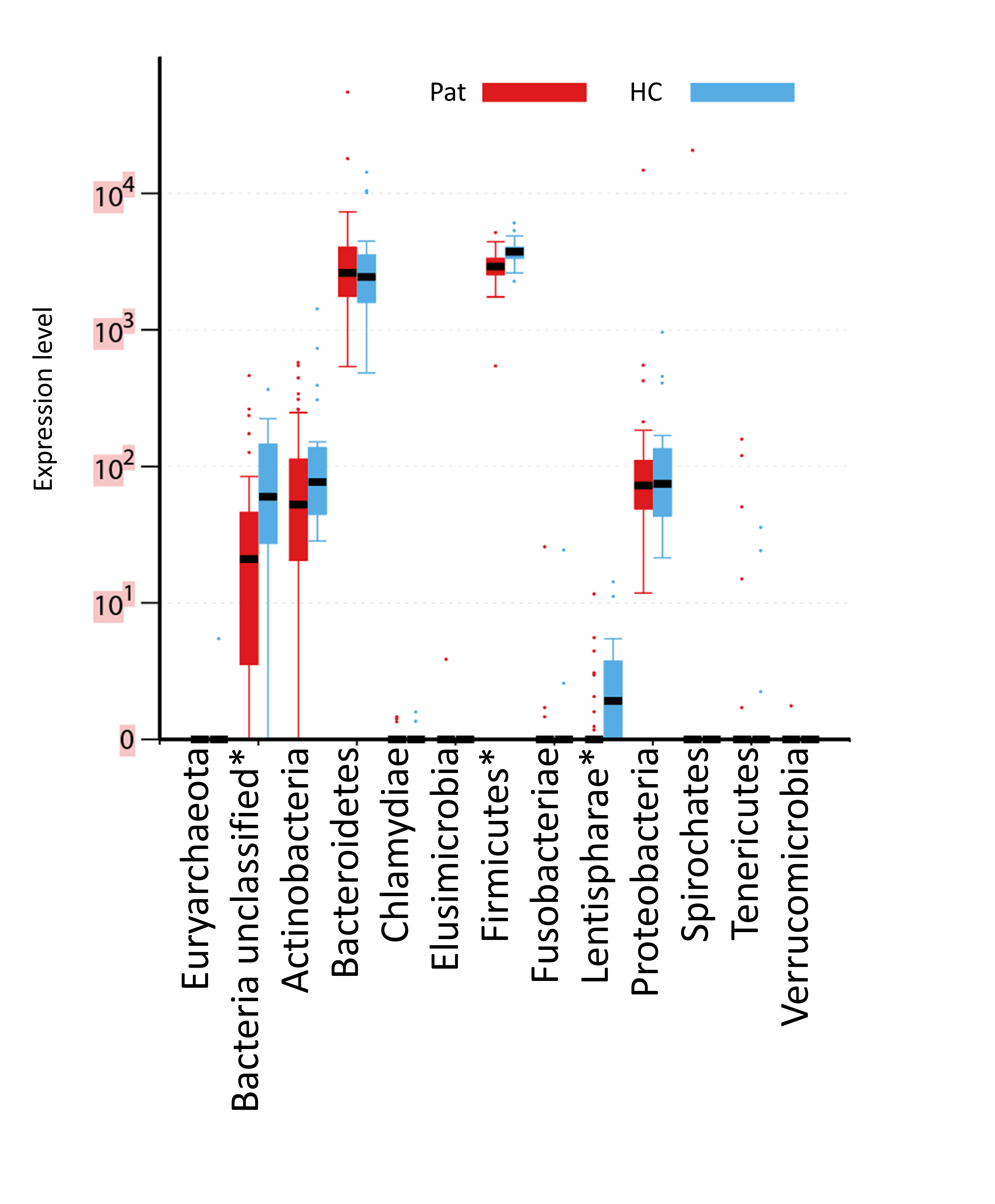


Supplementary Figure 1: Distribution of microbiota OTU counts in IBS patients and HC.

*statistically significant difference between the groups based on **Mann-Whitney** test, all p<0.01).

# Supplementary Figure 2


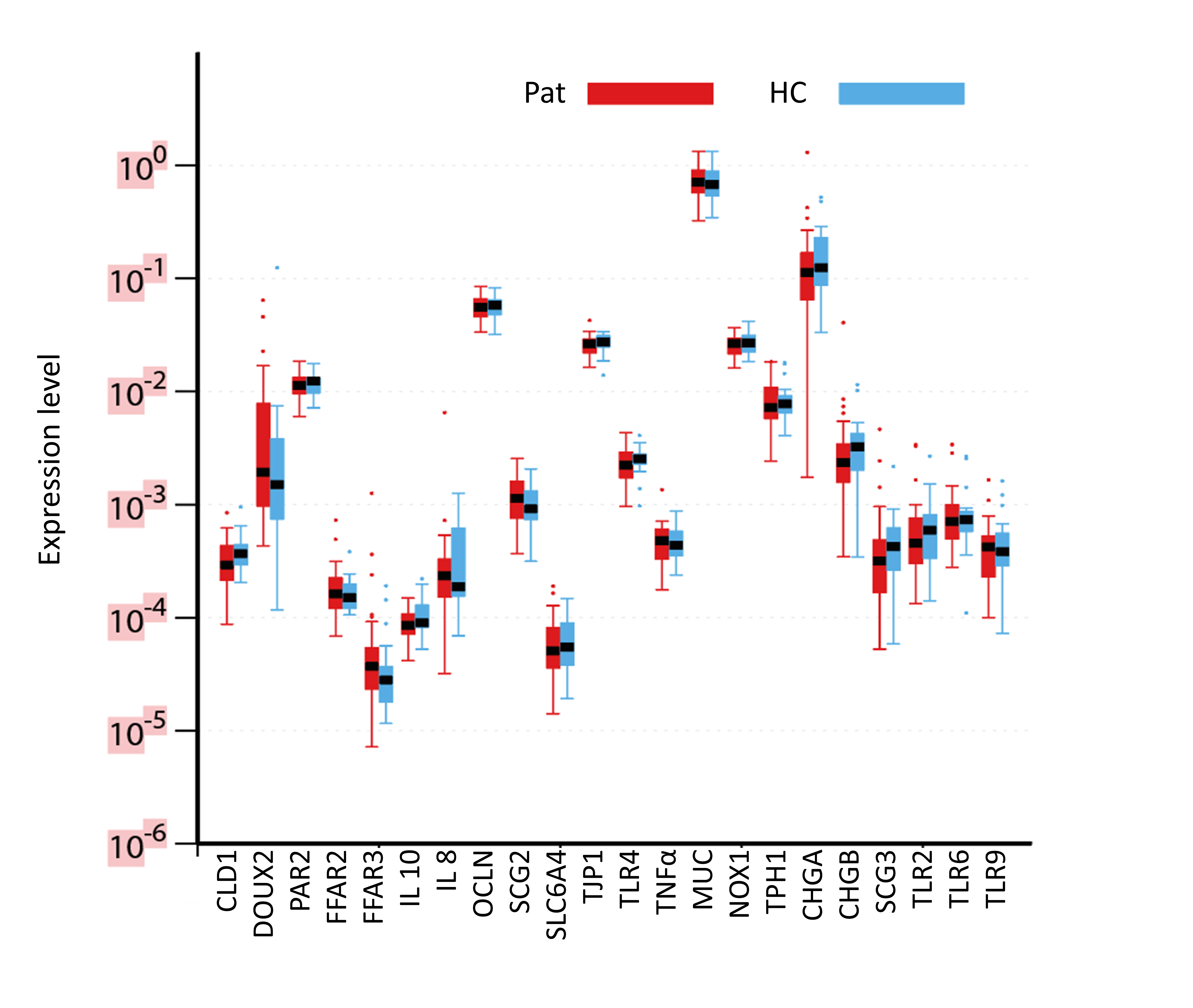


Supplementary Figure 2: Distribution of mucosal target gene expression in IBS patients and HC.

# Supplementary Table 1

Legend to Figure 5


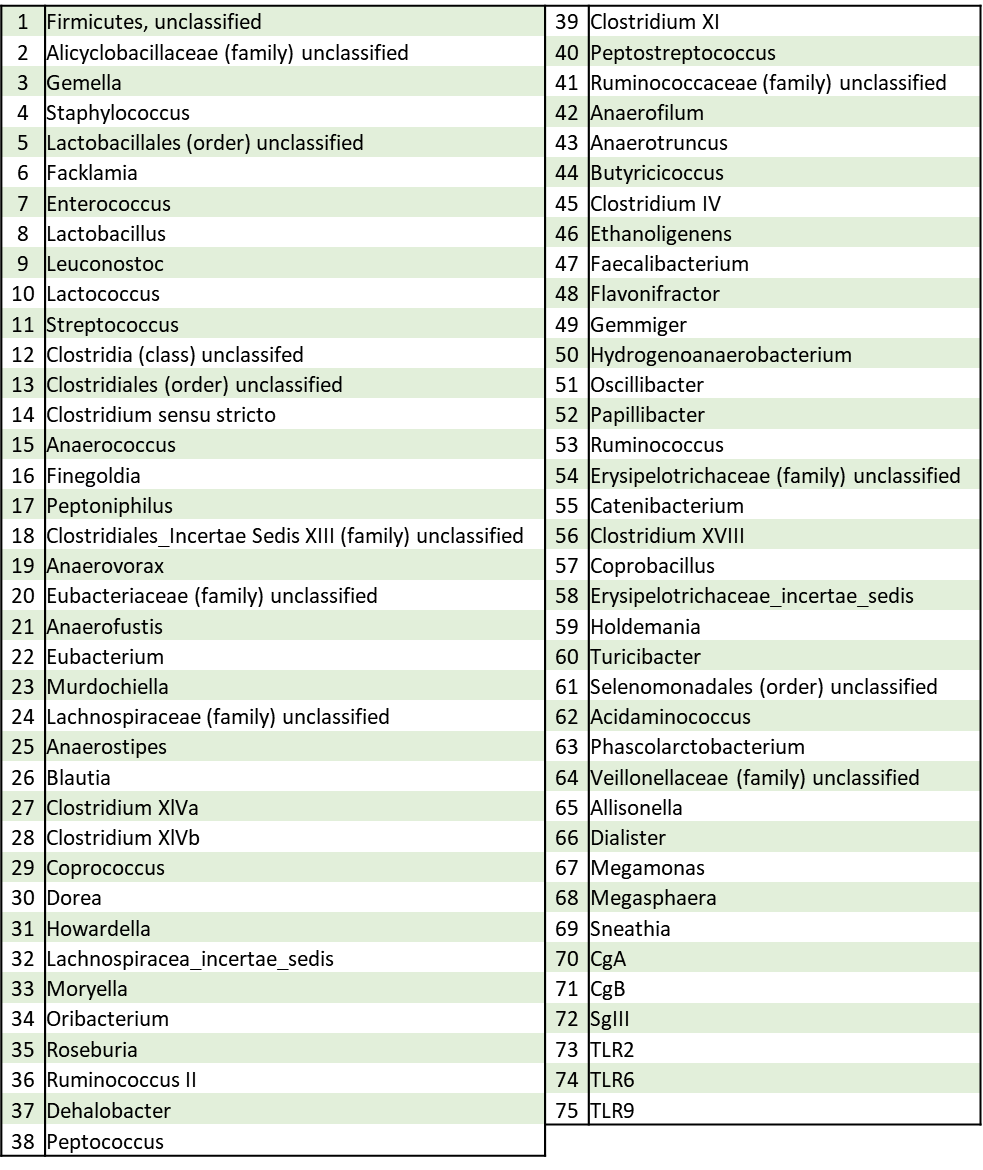


# Supplementary Table 2

Correlation coefficients to Figure 3, IBS patients. Statistically significant correlations (Spearman's Rank Correlation) are highlighted in color.





# Supplementary Table 3

Correlation coefficients to Figure 3, HC. Statistically significant correlations (Spearman's Rank Correlation) are highlighted in color.


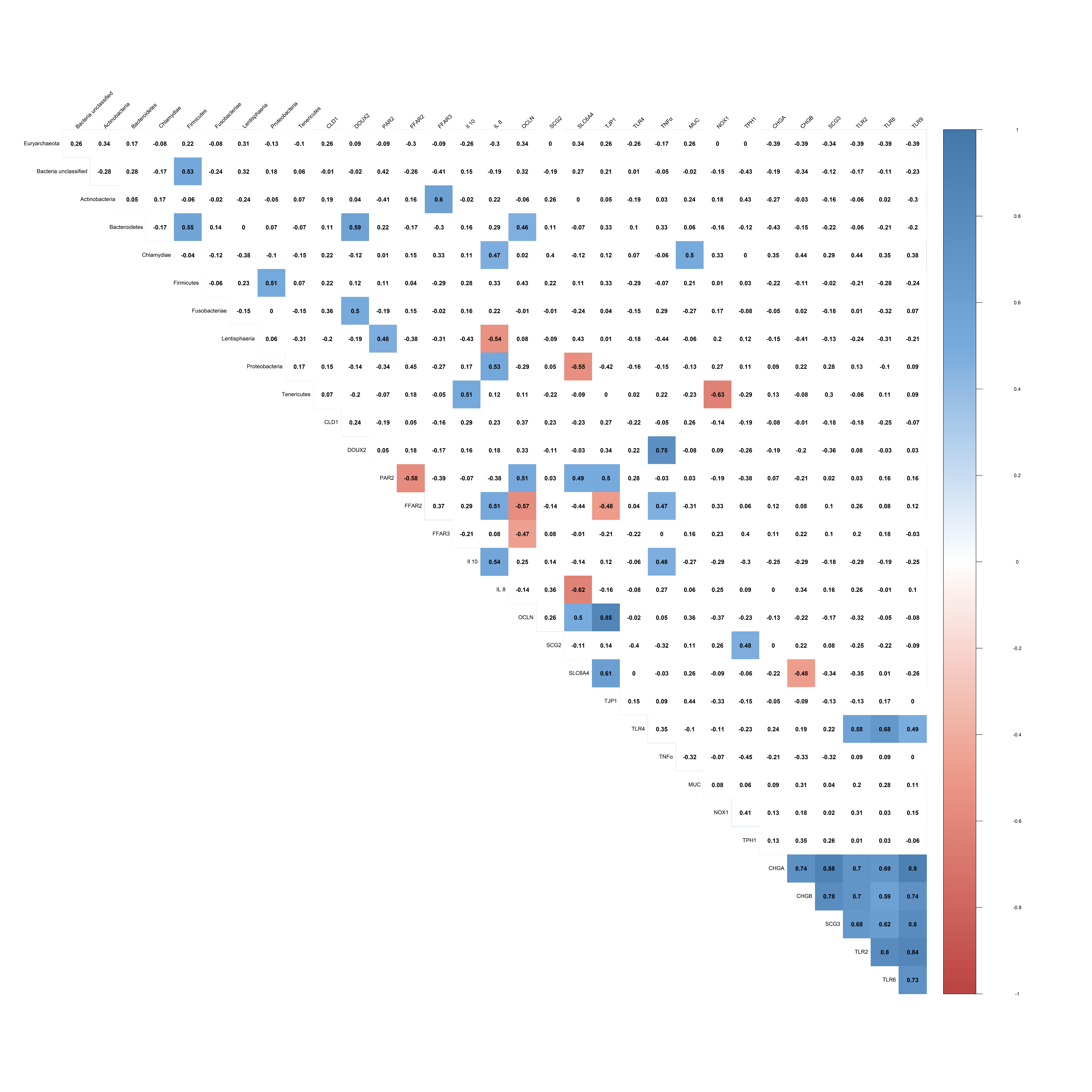


Supplementary Table 4

Correlation coefficients to Figure 5, statistically significant correlations (Spearman's Rank Correlation) are highlighted in color.


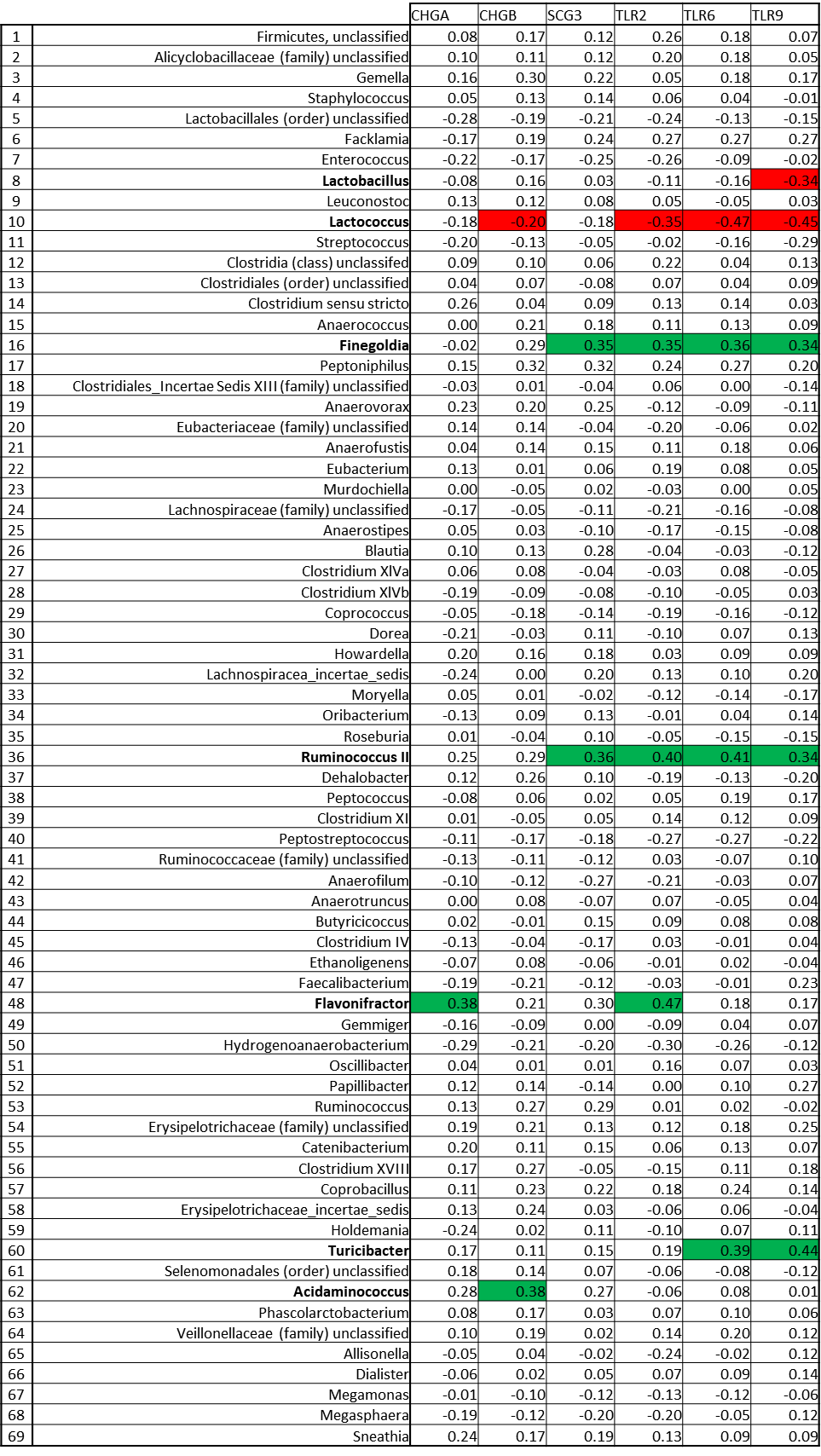


Positive correlation: Green

Negative correlation: Red

#
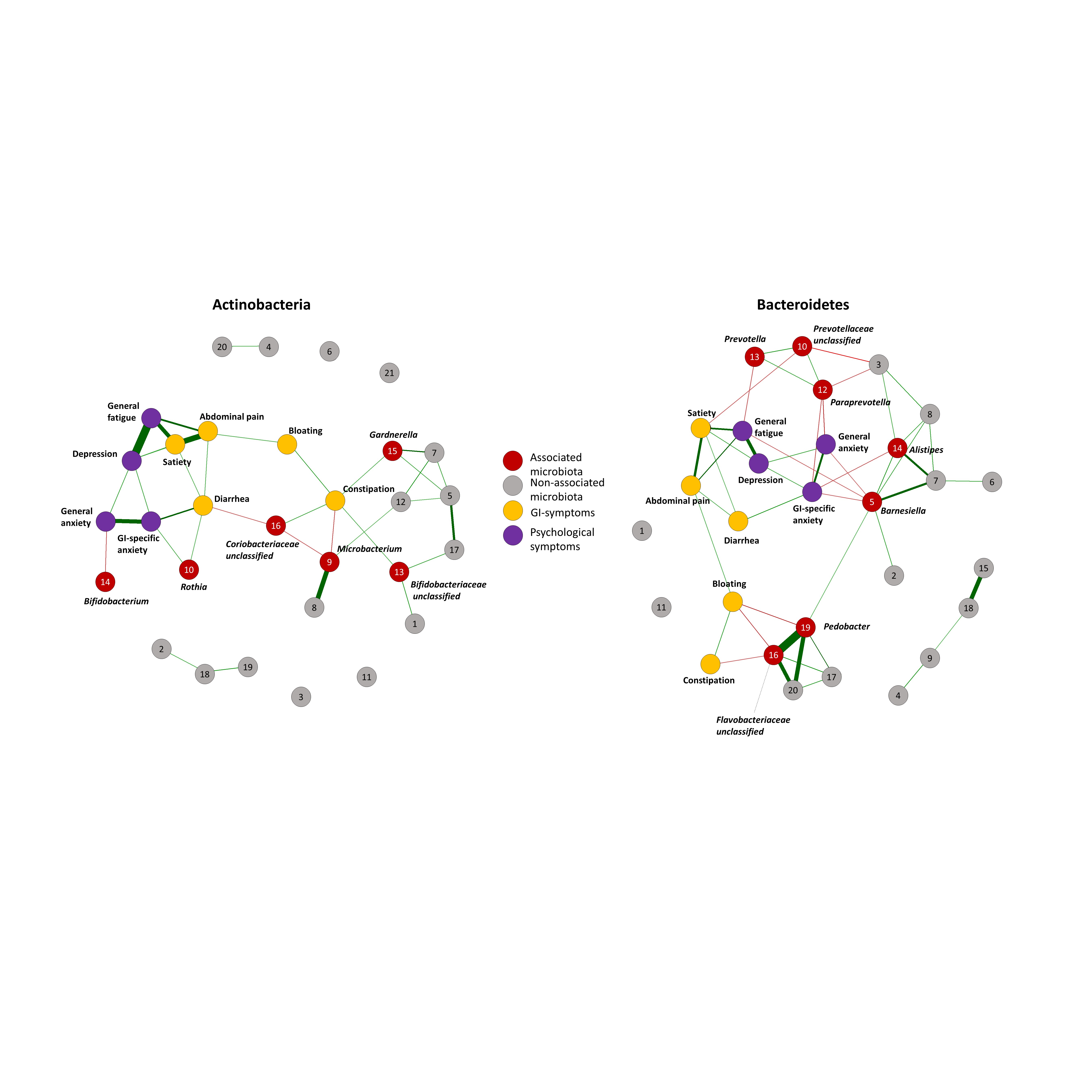
Supplementary Figure 3

Supplementary Figure 3: Intercorrelation networks of selected genus-level microbiota and symptoms. Only statistically significant correlations are plotted. The corresponding legend is given in Supplementary Table 5, and correlation coefficients are given in Supplementary Tables 6 and 7.

# Supplementary Table 5

Legend to Supplementary Figure 3

# Supplementary Table 6

Correlation coefficients ((Spearman's Rank Correlation, pairwise complete cases) to Supplementary Figure 3, Actinobacteria. Statistically significant positive correlations are highlighted green, negative correlations in red.

# Supplementary Table 7

Correlation coefficients (Spearman's Rank Correlation, pairwise complete cases) to Supplementary Figure 3, Bacteroidetes. Statistically significant positive correlations are highlighted green, negative correlations in red.
